# Lycopene protects sperm from oxidative stress in the experimental varicocele model

**DOI:** 10.1002/fsn3.2632

**Published:** 2021-10-17

**Authors:** Atefeh Babaei, Reza Asadpour, Kamran Mansouri, Adel Sabrivand, Siamak Kazemi‐Darabadi

**Affiliations:** ^1^ Department of Clinical Sciences Faculty of Veterinary Medicine University of Tabriz Tabriz Iran; ^2^ Medical Biology Research Center Health Technology Institute Kermanshah University of Medical Sciences Kermanshah Iran

**Keywords:** antioxidant activity, DNA damage, lycopene, oxidative stress, varicocele

## Abstract

Oxidative stress (OS) is an important parameter in the evaluation of infertility caused by varicocele. Antioxidants are the most commonly prescribed drugs in these patients. Lycopene molecule, as the powerful antioxidant in the carotenoid family, has beneficial effects on improving fertility in males. Therefore, we investigated the effects of lycopene on induced OS by varicocele in an animal model. Forty‐five adult male Wistar rats were divided into two groups: control (*n* = 12) and varicocele (*n* = 33). Two months after induced varicocele, five rats in each group were sacrificed randomly and induced varicocele was investigated. Remained rats were divided into five groups (*n* = 7), including the control (I), varicocele (II), varicocele reserving solvent (III), varicocele reserving lycopene 4 mg/kg (IV), and 10 mg/kg (V) for two months. At the end of the experiment, intracellular reactive oxygen species (ROS), malondialdehyde (MDA), total antioxidant capacity (TAC), %DNA damage, and antioxidant enzymatic levels were measured. The results indicated that there were significant increases in the levels of ROS, MDA, DNA damage, superoxide dismutase (SOD), sperm concentration, and motility in the varicocele groups compared with the control group. In the lycopene group (10 mg/kg), sperm concentration, the levels of TAC, and catalase (CAT) activity were improved so the levels of ROS, MDA, and %DNA damage were reduced compared with varicocele group. Our findings indicated that the administration of lycopene especially at a dose of 10 mg/kg in the varicocele group could protect sperm from OS and sperm DNA damage by increasing antioxidant activity and reducing ROS.

## Graphical abstract

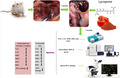

## 1 INTRODUCTION

Varicocele is defined as an abnormal dilatation and venous enlargement of the scrotal pampiniform plexus (Yetkin & Ozturk, 2018). Varicocele disease prevalence rates are about 35% and 80% with primary infertility and secondary infertility, respectively among men, and account for 35% of the total cases of infertility among couples (Alsaikhan et al. 2016). Several articles have demonstrated that varicocele has done negative effects on the concentration, viability, motility, and morphology of sperm (Hauser et al., 2001; Park et al., 2018). According to the current literature data, high levels of disorder in sperm plasma membrane integrity, sperm DNA fragmentation, and testicular germ cell apoptosis have been found in varicocele disease (Ammar et al., 2020). The pathophysiological mechanisms of varicocele that lead to infertility induction are not yet fully understood (Hassanin et al., 2018), but oxidative stress (OS), heat stress, hypoxia, hormonal imbalances, and exogenous toxicants affect the pathogenesis of varicocele (Agarwal et al., 2012). Existing evidence indicates that, even though men with varicocele have normal parameters of sperm, the damaged sperm DNA increases in these patients (Smith et al., 2006). To understand the mechanism of varicocele‐related DNA damages, many studies have focused on the OS system as a possible pathway that causes dysfunction in sperm of men with varicocele (Ammar et al., 2020; Jeremias et al., 2021; Manente et al., 2015; Saleh et al., 2003).

High levels of the reactive oxygen species (ROS) have a direct correlation with a decrease in spermatozoa count, motility, and DNA integrity and fertilization (Agarwal et al., 2014). ROS targets cell membranes and increases the peroxidation of membrane polyunsaturated fatty acids (PUFAs) (Cho et al., 2016). Malondialdehyde (MDA) is formed as an end product of the peroxidation of lipids, and MDA levels are commonly known as markers of OS and antioxidant status (Gaweł et al., 2004). Another important site of activity of ROS is the nuclear and mitochondrial DNA of sperm. High levels of ROS overwhelm the protective mechanisms and enzymes in sperm and oocyte that repair damaged DNA (Agarwal et al., 2003). Accordingly, the ROS/OS test can be used as additional diagnostic and prognostic information to provide treatment strategies in infertile men with varicocele (Cho et al., 2016).

Medical management, including the administration of antioxidants, can be a potential low‐risk solution to reduce OS in varicocele‐induced infertility (Garg & Kumar, 2016). However, antioxidant drug therapy for varicocele‐related infertility suffers from a lack of well‐conducted studies that could provide high evidence (Garg & Kumar, 2016). This problem stems from an unspecified treatment goal, poorly designed studies, inadequate measures, and various drug combinations (Garg & Kumar, 2016).

One of the natural powerful antioxidants is lycopene that is a red‐pigmented polyunsaturated molecule from the tetraterpene carotenoid family (Atasoy, 2012; Rao et al., 2006). Thirteen linear double bonds in the lycopene molecule make it the strongest anti‐radical compound in the carotenoid family and visualize its biological function (Hedayati et al., 2019). It has been shown that lycopene is twice as effective as β‐carotene and 10 times as effective as α‐tocopherol (Durairajanayagam et al., 2014). It is lipophilic and its sources include tomatoes, papayas, watermelons, apricots, pink grapefruits, and rosehips (Durairajanayagam et al., 2014). Lycopene accumulates in the testes, prostate, adrenal glands, and liver (Durairajanayagam et al., 2014; Rao et al., 2006). The concentration in the testes is 10 times higher than in other tissues, which may be due to a large number of lipoprotein receptors, relatively higher lipoprotein uptake, or higher metabolic/oxidation (Erdman Jr, 2005; Schmitz et al., 1991). The uneven distribution of lycopene indicates its biological role in the testes (Durairajanayagam et al., 2014). Several studies have shown that lycopene can protective sperm from OS by reducing ROS levels and increasing antioxidant enzyme levels (Durairajanayagam et al., 2014; Tripathy et al., 2020). They explained that this performance of lycopene reduces sperm DNA fragmentation and membrane lipid peroxidation (LPO) and finally improves concentration, motility, viability, and morphology in sperm of humans (Lu‐Lu & Zhi‐Gang, 2020; Williams et al., 2020) and animals (Tripathy et al., 2020; Tvrdá et al., 2016; Tvrda et al., 2017).

As a result, it is necessary to conduct more studies with a validated test design of lycopene with different doses to identify the effects of lycopene on OS in varicocele patients. In this study, we investigated the effects of lycopene on the OS induced by experimental varicocele via the measurements of intracellular ROS, MDA, total antioxidant capacity (TAC), %DNA damage, and antioxidant enzyme levels.

## 2 MATERIAL AND METHOD

### 2.1 Animals

Forty‐five adult male Wistar rats (180–200 g) aged 7–8 weeks old were obtained from the animal house of Kermanshah University of the Medical Sciences (Kermanshah, Iran) and were kept under standard conditions of controlled light (12:12 hr light/dark) and temperature (22 ± 2°C) with free access to standard food and water. All care and surgery procedures were performed following the guidelines for the care and use of laboratory animals, and all experiments were approved by the Ethical Committee of Tabriz University (IR.TABRIZU.REC.1399.041).

### 2.2 Induction of varicocele

**FIGURE 1:**
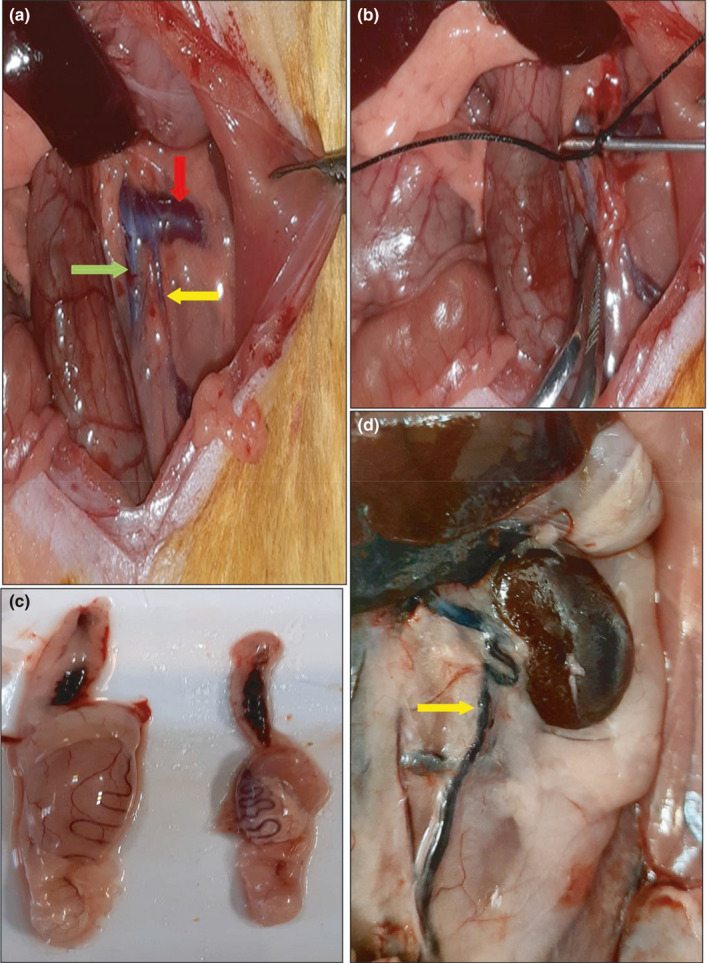
Induced varicocele in the rat. Normal vein (a), left renal vein have been shown with a red arrow, left spermatic vein with a yellow arrow, and the inferior vena cava with a green arrow. (b) Partial ligation of the left renal vein. (c) The testicles were decreased in size in the varicocele group (right side) in comparison with control groups (left side). (d) Distended testicular vein in varicocele (arrows)

Forty‐five rats were randomly divided into a control (healthy) group (*n* = 12) and a group that underwent surgery to induce varicocele (*n* = 33). Unilateral varicocele in the left testis was induced following intraperitoneal anesthesia with 75 mg/kg of 10% ketamine (Bremer pharma, Germany) and 5 mg/kg of 2% xylazine (2,320 Hoogstraten, Belgium). After disinfecting and shaving the abdominal surface, an incision (about 3–4 cm) was made from the midline of the abdominal cavity. After removing the internal organs and finding the left renal vein, a metal probe (0.8 mm in diameter) was placed parallel to the left renal vein. Around the left renal vein and metal, a wire probe was tied using a 0–4 silk suture at the nearest inferior vein (a and b parts of Figure 1). Approximately 50% narrowing was observed in the renal vessel. Then, the metal wire probe was removed gently. Finally, the abdominal surface muscles and skin were sutured separately using 0–3 silk sutures (Katz et al., 2014; Turner, 2001).

### 2.3 Experimental design

To confirm induced varicocele two months after the induction, five rats from the varicocele and control groups were sacrificed randomly with high doses of ketamine (*n* = 5). The concentration and motility of sperm, testis weight, % DNA damage, and % intracellular were measured to verify the induced varicocele. After confirming induced varicocele, 35 remained rats were divided into five groups (seven rats in each group).

1. Control group: healthy rats that received distilled water via gavage (2 ml, daily for 2 months).
2. Varicocele group: rats with varicocele that received distilled water via gavage (2 ml, daily for 2 months).
3. Solvent group: rats with varicocele that received solvent via gavage (2 ml with corn oil, daily for 2 months).
4. Lycopene group: rats with varicocele that received suspended lycopene in corn oil via gavage (Tinab Shimi, 92%–94%, T50206508, Mashad, Iran) (4 mg/kg, daily for 2 months).
5. Lycopene group: rats with varicocele that received suspended lycopene in corn oil via gavage (10 mg/kg, daily for 2 months).

**FIGURE 2:**
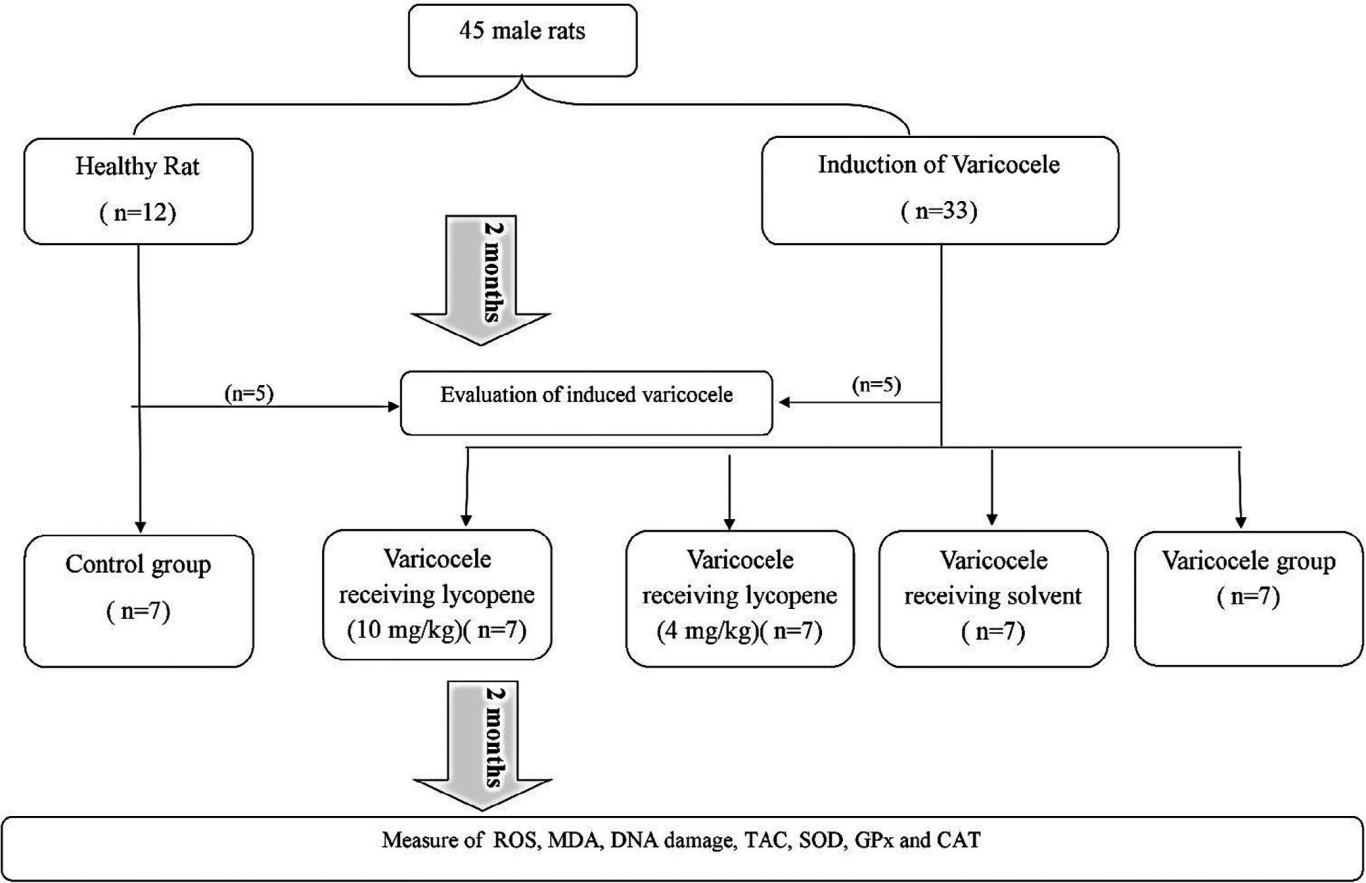
A flow chart of the study design. ROS: Intracellular reactive oxygen species, MDA: malondialdehyde, TAC: total antioxidant capacity, SOD: superoxide dismutase, GPx: glutathione peroxidase, and CAT: catalase

The flowchart of the designed experimental was shown in Figure 2.

### 2.4 Sperm collection

Four months after varicocele induction (2 months after treatment with lycopene), all the rats were sacrificed. The left caudal epididymides were carefully separated from the testis and minced in 5 ml of the mHTF (modified human tubule fluid) (HTF +HEPES, Avayeh Tejarat Aiyeh) containing 4 mg/ml of BSA (bovine serum albumin) in a 35‐mm plastic dish at 37°C. Then, epididymides were cut about 10 times using minimize scissors and incubated at 37°C under 5% Co_2_ for 5 min (Aoto et al., 2011). The left testis was removed and washed with normal saline and weighed.

### 2.5 Concentration and motility of sperm

Of 10 μl sperm suspension was diluted with 10 μl distilled water. Then, 10 μl of each sample was transferred into a hemocytometer. The concentration of spermatozoa was counted under a light microscope (Olympus) at ×200 magnifications (million/ml). The percentage of sperm motility was measured by placing 5 µl of the sample on a 37°C slide and counted at least 200 spermatozoa in 10 randomly selected fields under a light microscope (Seed et al., 1996).

### 2.6 DNA damage

One smear of each sperm sample (20 μl) was prepared on a slide and dried in dark. The slides were fixed in fresh Carnoy's solution (methanol: acetic acid, 3:1) overnight at −4°C. The slides were then washed with PBS (Phosphate‐buffered saline ) and stained with 0.19 mg/ml of fresh Acridine orange (AO) (Sigma, A6014‐10G) (10 ml of 1% AO +40ml of 0.1 M citric acid +2.5 ml of 0.3 M Na_2_HPO_4_.7H_2_O, pH = 2.5) in the dark for 10 min. After washing, the slides were immediately evaluated under a fluorescent microscope (450–490 nm). Two hundred sperm cells were counted on each slide. Spermatozoa with normal DNA had a spectrum of green fluorescence, and damaged DNA had a spectrum of orange to red fluorescence (Abbasi et al., 2011).

### 2.7 Intracellular ROS

Intracellular ROS was measured using 2’,7**’‐**dichlorodihydrofluorescein diacetate (DCFH‐DA) (Sigma‐Aldrich, 4091–99–0) a permeable stain in cell membranes and a probe specific for H_2_O_2_. The number of 1–2 × 10^6^ sperms/ml in PBS (pH 7.4, 37°C) was added to 5 μM of DCFH‐DA and incubated at 37˚C in the dark for 30 min. Then, the cell suspension was centrifuged at 37°C (500*g*, 3 min), and the supernatant was removed in the dark. Cells were re‐suspended in 1 ml of PBS (37°C) and analyzed by a fluorescence detector‐1 (FL‐1) (Attune™ NxT Flow Cytometer, Thermo Fisher Scientific) (488–530 nm) (Ata‐Abadi et al., 2020). CellQuest version 2.9 was used for data analysis. The mean fluorescence intensity of the analyzed sperm cells was determined after gating the cell population using forward and side scatter signals. Every test sample was normalized against an unstained control sample (Du Plessis et al., 2010).

### 2.8 Lipid peroxidation

The left testes tissue samples were removed and stored at −80°C for biochemical tests. At the time of assay, after thawing the tissues, 0.6 g of each sample was homogenized in 50 mM of PBS (Freiberger et al., 2004). MDA levels are an indicator of the severity of oxidative stress. Evaluation of testicular MDA content is based on the reaction of thiobarbituric acid (TBA) with MDA, which produces a colored product that can be measured at 532 nm (Hassani‐Bafrani et al., 2019). MDA levels were measured using a commercial standard kit (Nalondi^™^ Navand Salamat) by spectrophotometry (UV‐2601, Rayleigh) and were reported in nmol/mg protein.

### 2.9 Total antioxidant capacity

The total antioxidant capacity (TAC) was measured using the ferric reduction antioxidant power (FRAP) method (Benzie & Devaki, 2018) by a commercial standard (Naxifer^™^) by spectrophotometry (UV‐2601, Rayleigh) at 593 nm. The results of TAC are expressed in nmol/mg protein.

### 2.10 The activity of antioxidant enzymes

The tissue levels of superoxide dismutase (SOD), glutathione peroxidase (GPx), and catalase (CAT) were assayed using commercial standard kits (Nasdox^™^, Nagpix^™^, and Nactaz^™^ Navand Salamat). The absorbance rates were recorded by a spectrophotometer (UV‐2601, Rayleigh) at 405, 340, and 550 nm for SOD, GPx, and CAT, respectively. The results of SOD, GPx, and CAT are reported in U/mg protein, nmol/mg, and nmol/mg protein respectively (Benzie & Devaki, 2018). The protein content was assayed based on the Bradford method (Kruger, 2009) by a commercial standard kit (Nadford^™^, Navand Salamat).

### 2.11 Statistical method

All data were analyzed using the SPSS software version 22.0, and the Shapiro–Wilk test was used to assess the normal distribution. All the data showed normal distribution, and differences within groups were compared by one‐way analyses of variance (ANOVA) using a post hoc test (Duncan). To verify induced varicocele, differences between the two groups (control and induced varicocele) were analyzed by an independent sample *t* test. Collected data are presented as mean ±standard error of the mean (*SEM*), and *p* < .05 was considered to be significant.

## 3 RESULTS

### 3.1 Induced varicocele

To confirm induced varicocele, five rats were sacrificed randomly two months after the surgery and showed an increase in the diameter of left internal spermatic veins by about 2 times or more. The varicose veins of the left spermatic vein and the reduced testes size were visible in varicocele rats (Figure 1d). The testis weight increased in the varicocele group compared with the control group (*p* < .05, Figure 1c). Sperm parameters including concentration and motility were lower, and % ROS intracellular were higher in induction varicocele group compared with the healthy rats (*p* < .05), (Table 1).

**TABLE 1:** Testes weight (g), concentration (*10^6^), % motility, % DNA damage, and % ROS after two months of varicocele induction

% ROS	%DNA Damage	Motility%	Concentration (*10^6^)	Testis weight (g)	Groups
23.88 ± 3.24^b^	5.00 ± 0.47^b^	76.80 ± 5.13^a^	112.00 ± 6.51^a^	0.98 ± 0.05^a^	Control
56.67 ± 15.0^a^	10.40 ± 1.31^a^	62.70 ± 4.51^b^	84.80 ± 7.05^b^	0.7 5 ± 0.07^b^	Varicocele
0.065	0.005	0.073	0.022	0.029	Sig.

Note

All data are given as mean ± *SEM* (*n* = 5). a and b present the significant differences (*p* < .05) between differently marked data.

### 3.2 Sperm parameters

After two months of treatment, the concentration of sperm in the varicocele, solvent, and varicocele with lycopene (4mg/kg) groups significantly decreased compared with the control group (*p* < .05). The concentration of sperm did not show significantly different between control group and varicocele with lycopene (10 mg/kg) group. According to the results, the control group showed the highest percentage of motility compared with the other groups (*p* < .05, Table 2).

**TABLE 2:** Effect of lycopene on testes weight (g), concentration (*10^6^), and %motility in varicocele rats after four months

Groups	Testis weight (g)	Concentration (*10^6^)	Motility%
Control	1.64 ± 0.04^a^	103.28 ± 6.63^a^	75.36 ± 5.57^a^
Varicocele	0.78 ± 0.12^c^	51.14 ± 10.50^b^	45.93 ± 6.50^b^
VCL‐Solvent	0.72 ± 0.95^c^	55.28 ± 11.09^b^	42.36 ± 5.38^b^
VCL+Ly4	1.09 ± 0.07^b^	63.86 ± 10.28^b^	49.43 ± 3.70^b^
VCL+Ly10	1.15 ± 0.09^b^	76.57 ± 12.08^ab^	54.00 ± 4.87^b^
Sig.	0.0001	0.008	0.001

Note

All data are given as mean ± *SEM* (*n* = 7). a and b present the significant differences (*p* < .05) between differently marked data.

### 3.3 DNA damage

**FIGURE 3:**
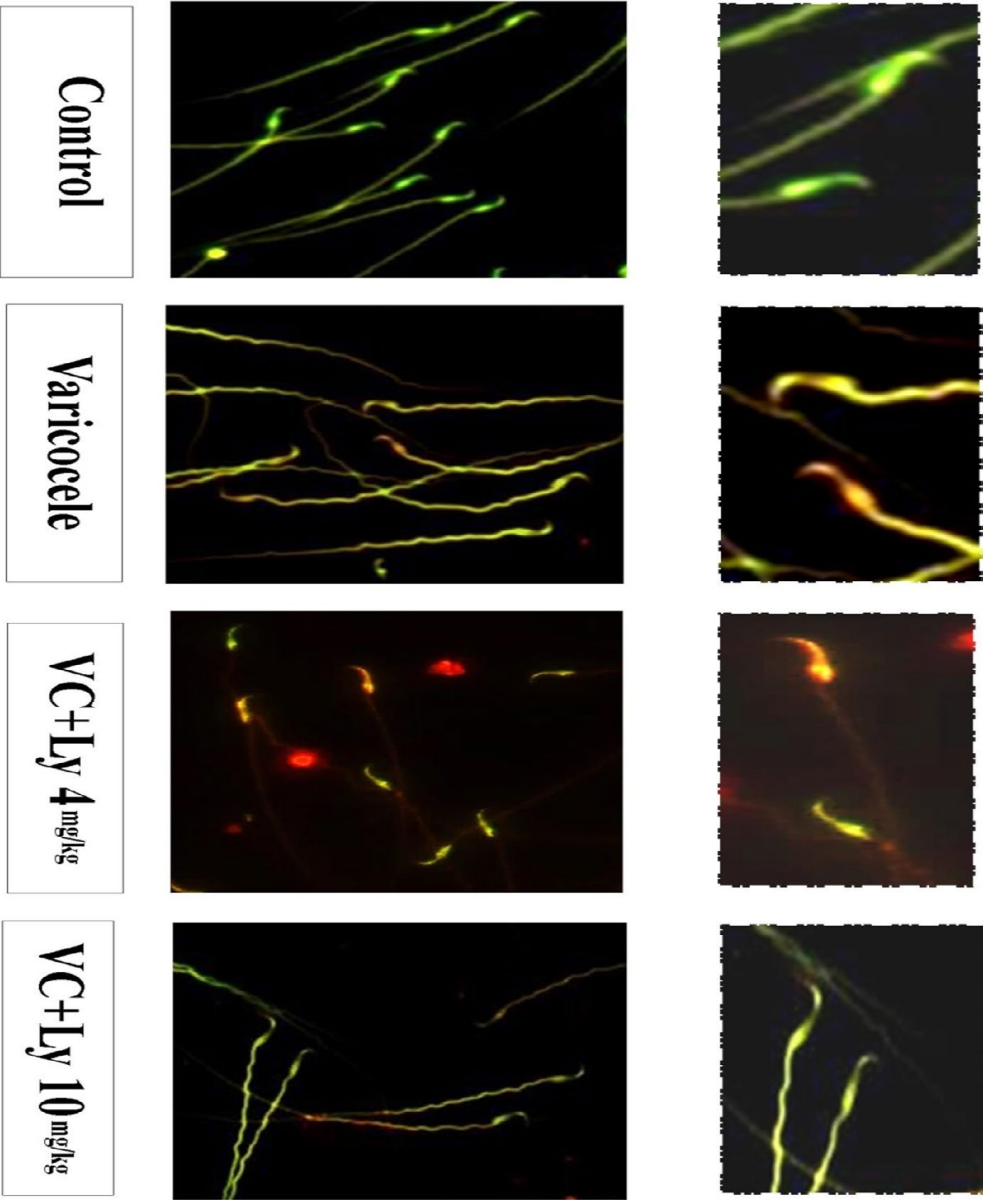
Acridine orange staining. Spermatozoa showing green fluorescence had normal DNA, whereas sperms showing a spectrum of red‐orange fluorescence had damaged DNA

**FIGURE 4:**
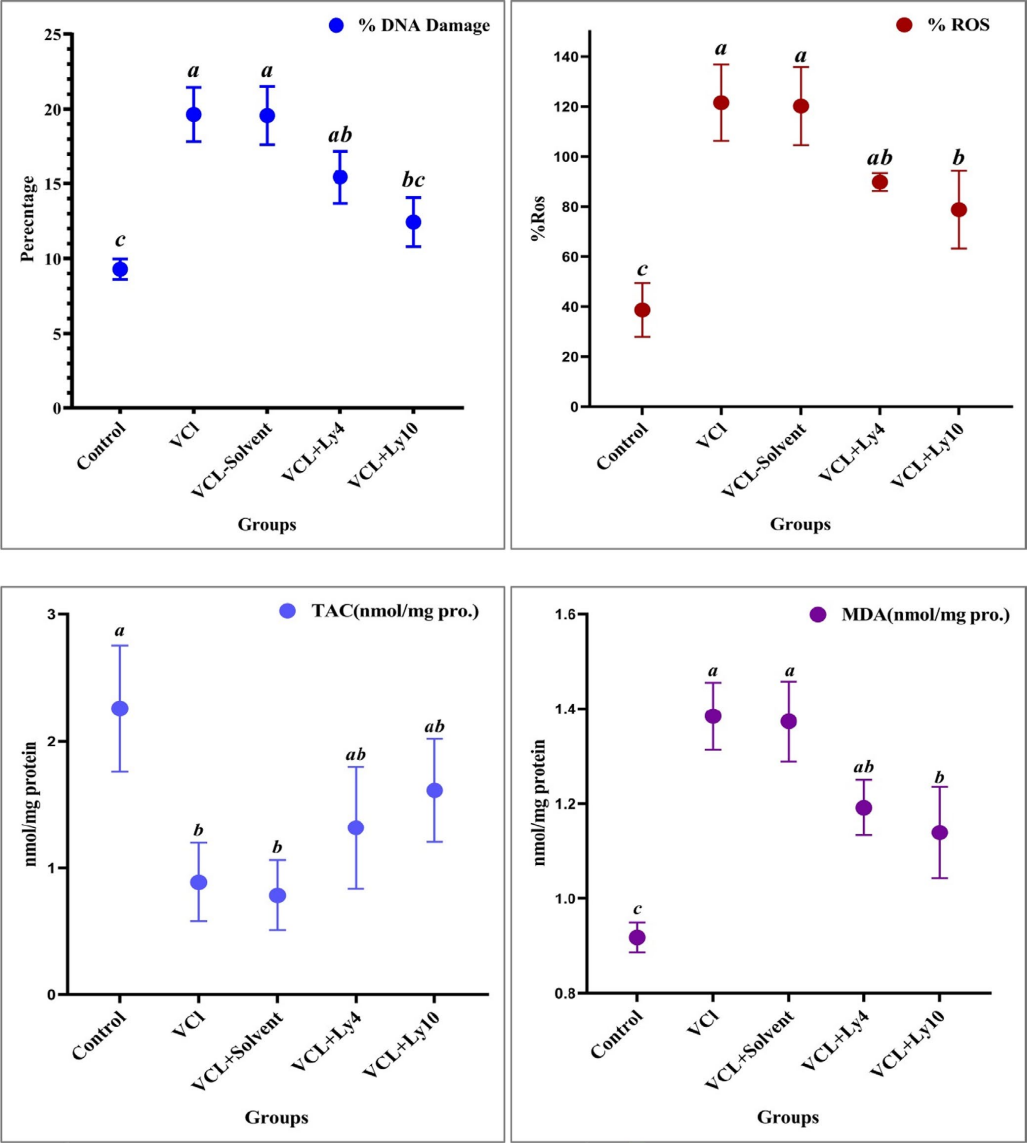
Effect of lycopene on % DNA damage, ROS (reactive oxygen species), malondialdehyde (MDA), and total antioxidant capacity (TAC) in varicocele rats. All data are given as mean ± *SEM*. a, b, and c present the significant differences (*p* < .05) between differently marked data

Acridine orange staining was used to assess sperm DNA damage. Spermatozoa with green fluorescence had normal DNA, and those with a spectrum of orange to red fluorescence had damaged DNA (Figure 3). The comparison outcomes of the DNA damage analysis in five groups are shown in Figure 4. Based on these results, DNA damages in the varicocele (19.64 ± 1.8) and varicocele reserving solvent (19.57 ± 1.93) groups significantly increased compared with the control (9.28 ± 0.68) and varicocele reserving lycopene (10 mg/kg) (12.43 ± 1.63) groups (*p* < .05). Our findings indicated that there was no statistically remarkable deferent between the control and lycopene (10 mg/kg) groups (*p* > .05).

### 3.4 Intracellular ROS

The levels of intracellular ROS in the sperm of varicocele, solvent, and varicocele reserving lycopene (4 and 10 mg/kg) groups remarkably increased in comparison with the control group (38.7 ± 10.79) (*p* < .05). However, a significant reduction was observed in the level of ROS in varicocele reserving lycopene (10 mg/kg) group (78.8 ± 15.58) compared with the varicocele (121.6 ± 15.25) and varicocele reserving solvent groups (120.22 ± 15.61) (*p* < .05, Figure 4).

### 3.5 Lipid peroxidation

The analyses of MDA levels represented a remarkable enhancement in varicocele (1.38 ± 0.07), solvent (1.37 ± 0.08), and lycopene (4 and 10 mg/kg) groups compared with the control group (0.92 ± 0.03) (*p* < .05). However, the administration of lycopene (10 mg/kg) led to a lower level (1.14 ± 0.09) of MDA than the varicocele group (*p* < .05, Figure 4).

### 3.6 Total antioxidant capacity

A significant reduction was observed in TAC level of testes in varicocele group compared with the healthy rats (Figure 4). On the other hand, the administration of lycopene (4 and 10 mg/kg) significantly increased TAC of testes (1.31 ± 0.48), (1.61 ± 0.40) compared with varicocele (0.89 ± 0.30) and solvent groups (0.78 ± 0.28) (*p* < .05). This study represented that there were no significant differences in the level of testes TAC between the control (2.26 ± 0.5) and lycopene groups (*p* > .05).

### 3.7 The activity of antioxidant enzymes

**FIGURE 5:**
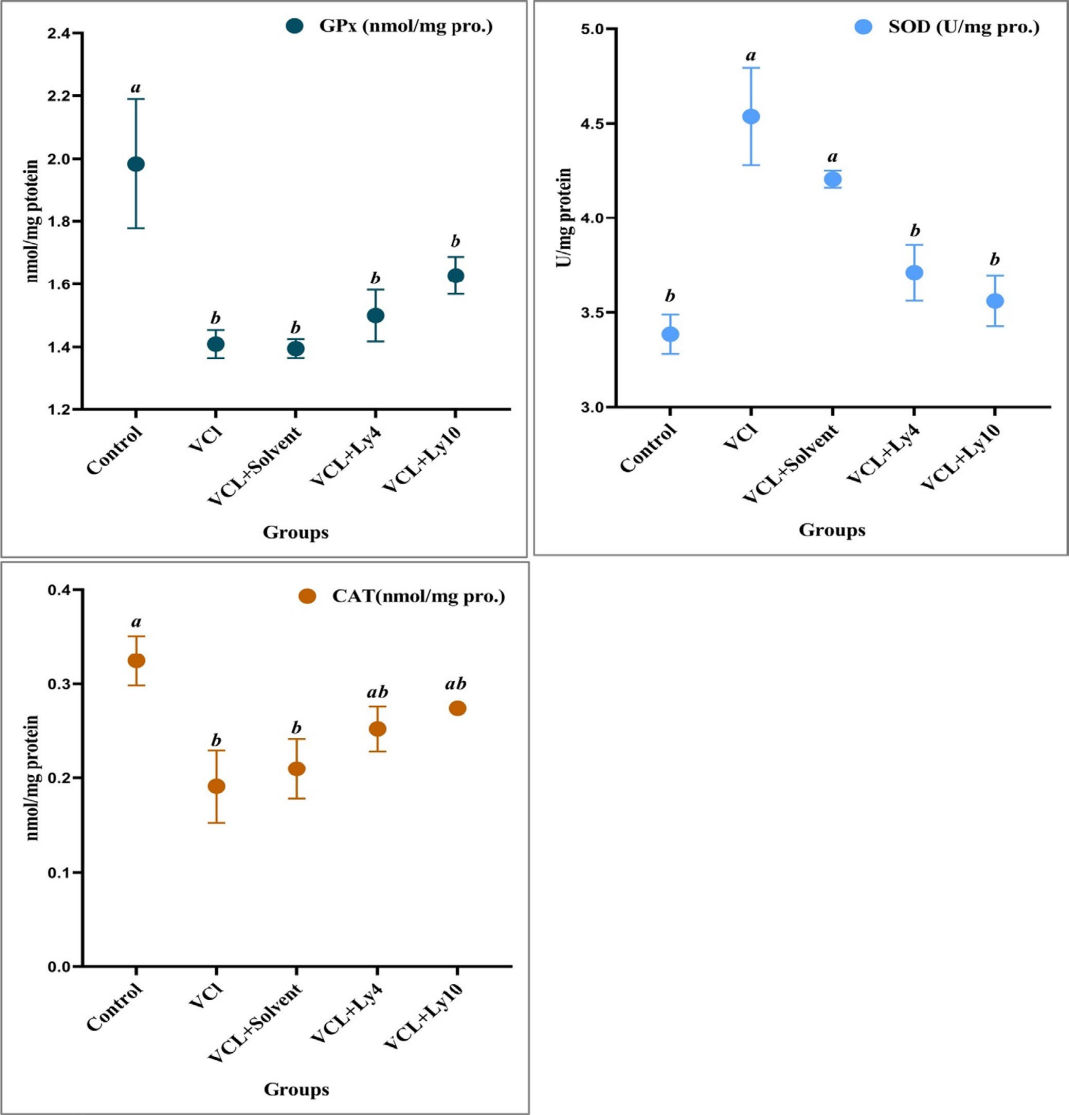
Effect of lycopene on the activity of glutathione peroxidase (GPx), superoxide dismutase (SOD), and catalase (CAT) in the testes of varicocele rats. All data are given as mean ± *SEM*. a, b, and c present the significant differences (*p* < .05) between differently marked data

We observed a significant decrease in the activity of catalase (CAT) in the varicocele (0.19 ± 0.04) and varicocele reserving solvent (0.21 ± 0.03) groups compared with the control group (0.32 ± 0.026) (*p* < .05), but these changes were not significant between lycopene groups (4 and 10 mg/kg, 0.25 ± 0.024 and 0.27 ± 0.005, respectively) and to control group (*p* > .05, Figure 5).

The level of GPx was highest in the control group in comparison with the other groups (*p* < .05, Figure 5).

Our results indicated that the activity of SOD in varicocele (4.54 ± 0.26) and varicocele reserving solvent (4.21 ± 0.045) groups significantly increased compared with control (3.38 ± 0.10) and lycopene groups (4 and 10 mg/kg, 3.7 ± 0.14 and 3.6 ± 0.13, respectively) (*p* < .05, Figure 5).

## 4 DISCUSSION

A growing body of investigations done on OS proposes that OS is one of the important factors leading to abnormal sperm parameters and subsequent infertility in men with varicocele (Agarwal et al., 2009). The negative impact of excessive varicocele‐induced ROS is not only limited to abnormal semen parameters but also increases sperm DNA fragmentation, which can lead to poor sperm function and poor fertilization results (Hassanin et al., 2018).

Testicular hyperthermia and hypoxia have an important role in OS‐induced testicular dysfunction in varicocele disease (Makker et al., 2009). Due to inadequate cell repair systems, spermatozoa have very little cytoplasmic content, and consequently, the insufficient antioxidant content is exposed to OS (Dutta et al., 2019). It is well known that varicocele has to correlate with excess ROS generation by spermatozoa, high rates of DNA damage in these cells, and depleted antioxidant levels in the seminal plasma (Agarwal et al., 2003; Hassanin et al., 2018; Jeremias et al., 2021; Razi et al., 2021; Saleh et al., 2003). This study elucidated that induced varicocele in rats enhanced OS. Our results showed a remarkable increase in the levels of ROS, LPO, and DAN damage in rats with varicocele (*p* < .05). Moreover, a significant decrease was observed in TAC, activity of antioxidant enzymes, sperm concentration, and motility in rats with varicocele (*p* < .05).

Appropriate amounts of ROS play an important role in normal sperm function, including hyperactivation, capacitation, acrosomal reaction, and finally oocyte fusion (Griveau & Lannou, 1997). An impaired balance between antioxidant capacity and ROS production results in the accumulation of oxidative products (Aitke et al., 2014). The measurement of both ROS and TAC is essential in the assessment of OS (Kashou et al., 2013). Like ROS, antioxidants can be sourced endogenously and exogenously that act as enzymatic and non‐enzymatic antioxidants. Enzymatic antioxidants catalyze ROS to neutral products, and non‐enzymatic antioxidants directly neutralize free radicals (Ritchie & Ko, 2021). Three predominant antioxidant enzymatic components in semen include SOD, GPX, and CAT (Adewoyin et al., 2017). Common ROS are superoxide anion (O^‐2^), hydroxyl radical (OH^•^), and the strong oxidizer hydrogen peroxide (H_2_O_2_) (Kashou et al., 2013). In the presence of SOD, O^‐2^ is converted to H_2_O_2_. It is a powerful membrane‐permeable oxidant that induces oxidative damage to lipids, proteins, and DNA in the cell and has to be rapidly eliminated from the cell (Aitken & Roman, 2009). It has been reported that SOD activity was negatively correlated with the sperm motility and capacity for oocyte fusion and positively associated with the induction of oxidative damage (Aitken et al., 1996). Spermatozoa are highly susceptible to the cytotoxic effects of H_2_O_2_, and the elimination of H_2_O_2_ is either affected by CAT or GPX (Razi & Roman, 2009). GPX, with significant concentrations in both the mitochondrial matrix and nucleus, maintains mitochondrial function and protects DNA by reducing H_2_O_2_. CAT also counteracts OS by converting H_2_O_2_ to O_2_ and H_2_O (Peltola et al., 1992).

Our results showed that with decreasing testicular antioxidant capacity and increasing ROS, SOD activity increased in the varicocele group to balance between ROS and H_2_O_2_ levels. In addition, the levels of MDA and % DNA damage were highest, and sperm concentration and motility were lowest in the varicocele group, which confirms the destructive effect of excessive ROS in increasing membrane LPO and consequent DNA damage in varicocele patients (Alsaikhan et al., 2016; Ammar et al., 2020; Cho et al., 2016; Jeremias et al., 2021; Peltola et al., 1992; Saleh et al., 2003; Smith et al., 2006).

Interestingly, the results revealed that the administration of lycopene in the varicocele group, especially at a dose of 10 mg/kg, reduced the ROS levels by increasing both TAC levels and CAT enzyme activity. Finally, reduced DNA damage and LPO and finally an increase in sperm concentration were observed in this group compared with varicocele groups.

Several studies have been performed on the mechanism of lycopene to reduce the risk of chronic diseases caused by OS such as cancer (Song et al., 2021), osteoporosis (Rao et al., 2020), hypertension (Bose & Agrawal, 2007), neurodegeneration (Saini et al., 2020), and cardiovascular disease (Petyaev et al., 2019). These mechanisms include oxidative and non‐oxidative mechanisms (Durairajanayagam et al., 2014). Lycopene has 11 conjugated double bonds (Atasoy, 2012); hence, it contains many electrons that can donate to free radicals such as O^‐2^, H_2_O_2,_ NO, OH^•^, and thus neutralize them (Krishnamoorthy et al., 2011). As an antioxidant, lycopene reduces the burden of ROS and thus OS, thereby preventing oxidative damage to lipids, proteins, and DNA (Palozza et al., 2012). Furthermore, lycopene is lipophilic and tends to accumulate in cell membranes and lipoproteins, thus, directly neutralizing ROS by acting as a singlet oxygen quencher, hence causing the overall amount of ROS to decrease (Durairajanayagam et al., 2014). Additionally, lycopene may have other beneficial effects via non‐oxidative mechanisms in the testis, such as gap junction communication, modulation of gene expression, regulation of the cell cycle, and immunoenhancement (Palozza et al., 2012).

In 2020, a study has been conducted on the effect of lycopene at a dose of 1 mg/kg intraperitoneally on varicocele in rats (Antonuccio et al., 2020). They reported that treatment with lycopene significantly increased weight of testes and decreased MDA in rats with varicocele. The design of this study is unclear and, on the other hand, varicocele in the contralateral testis was considered as the control for varicocele‐operated testis in this study (Antonuccio et al., 2020). The unilateral varicocele induction in rats affected both left and right testicles simultaneously (Razi et al., 2011).

Various studies investigated the effect of lycopene on fertility in men and animals and showed promising results. Our results clearly showed that lipid peroxidation, total antioxidant capacity, the level of catalase, and SOD improved in varicocele‐treated rats with lycopene especially at a dose of 10 mg/kg. Tripathy et al. (2020) investigated the direct role of lycopene on cyproterone acetate (CPA)‐induced infertility in rat. They showed antioxidant enzyme activities such as catalase, peroxidase, SOD, and GST were recovered after direct exposure of lycopene to the CPA‐treated (CPA + lycopene‐treated) infertile animals may be due to the potent antioxidant activity of lycopene either by rapid destruction of free radical (Tripathy et al., 2020).

This study illustrated that admiration of lycopene in varicocele rats decreased the ROS level and lipid peroxidation and protected sperm from DNA damage. A study about antioxidant effects of lycopene on bovine sperm and oxidative profile following cryopreservation showed that lycopene exhibited significant reactive oxygen species‐trapping and antioxidant properties which may prevent oxidative damage to frozen‐thawed sperm, and, thus, decreased lipid peroxidation and oxidative DNA damage (Tvrda et al., 2017).

Similar to our results, Gupta & Kumar (2002) illustrated that oral lycopene therapy seems to have a role in the management of idiopathic male infertility and maximum improvement seems to occur in the sperm concentration (66% cases, Gupta & Kumar, 2002).

Other studies also explained that lycopene reduces sperm DNA fragmentation, LPO of the plasma membrane, and improves concentration, motility, viability, and morphology in sperm of infertile man (Lu‐Lu & Zhi‐Gang, 2020; Mohanty et al., 2001; Williams et al., 2020) and animals (Bucak et al., 2015; Mangiagalli et al., 2010, 2012). According to previous studies and the present study on the effects of lycopene on infertility, lycopene can be used as an antioxidant supplement to reduce OS and its complications in varicocele patients.

## 5 CONCLUSION

In brief, the results of our study showed the high antioxidant effect of lycopene on reducing OS by varicocele. The administration of lycopene in the varicocele group, especially at a dose of 10 mg/kg, reduced the ROS level by increasing both TAC levels and TAC enzyme activity. Finally, reduced DNA damage and LPO have been observed in this group compared with varicocele rats. However, more research is needed to evaluate the effect of lycopene on the other pathophysiological mechanisms of induced infertility by varicocele disease.

## 6 RESERCH INVOLVING ANIMAL RIGHTS

All the animal procedures were performed in accordance with the guidelines for the care and use of laboratory animals, and all experiments were approved by the Ethical Committee of Tabriz University (IR.TABRIZU.REC.1399.041).

## CONFLICT OF INTEREST

The authors declared no potential conflicts of interest with respect to the research, authorship, and/or publication of this article.

## AUTHOR CONTRIBUTIONS

A.B. designed the study and involved in analysis and/or interpretation; R.A. and K. M. supervised the study and involved in funding; A.B, K. M, and R.A. involved in data collection and/or processing; A. S. and S. KD reviewed the literature.

## ETHICAL APPROVAL

All the authors have approved that the submitted works are original, and the paper has not been published and is not being considered for publication elsewhere.

## Data Availability

Datasets generated for this study are available on request to the corresponding author.
